# Left Over

**Published:** 1884-03

**Authors:** 


### LEFT OVER.

Considerable matter that had been prepared for this number, some of which was in type, has been crowded out. Editorials are always the first to give way, but we are obliged to ask the indulgence of some of our correspondents and contributors. Their articles shall have place at an early day.

